# Relationship Between Body Mass Index (BMI) and Depression Among the General Population of Al-Madinah Al-Munawwarah, Saudi Arabia

**DOI:** 10.7759/cureus.87686

**Published:** 2025-07-10

**Authors:** Mohammed Salah Alfahal, Ahmed M Alrehaili, Abdullah M Barry, Abdulelah A Alharoon, Ammar N Alraddadi, Ahmed K Alsaif, Yonus A Hawsawi, Mohammed K Alharbi, Abdulkarim M Alsani, Mohammed Elmuttalut

**Affiliations:** 1 Psychiatry, Al-Rayan College of Medicine, Madinah, SAU; 2 Medicine and Surgery, Al-Rayan National Colleges, Madinah, SAU; 3 College of Medicine, Qassim University, Unaizah, SAU

**Keywords:** adult, body mass index, depression, major depressive disorder, obesity

## Abstract

Background: Depression and obesity are recognized as major global health issues. Depression is described as persistent feelings of sadness and loss of interest. Obesity is characterized by excessive fat accumulation, with a body mass index (BMI) of ≥30 kg/m^2^. Both conditions significantly contribute to the number of incidents of disability, chronic illnesses, and reduced life expectancy.

Objectives: This study aimed to examine the relationship between obesity and depression among adults in Al-Madinah Al-Munawwarah, Saudi Arabia, and to explore their association with factors such as demographics, BMI, and the presence of chronic or psychiatric illnesses.

Methods: This cross-sectional study, conducted between August 2024 and March 2025, used an online survey asking about chronic diseases, prior diagnoses of mental health disorders, medication or supplement use for weight loss, height, weight, and depression severity, assessed via the Patient Health Questionnaire (PHQ-9). Data analysis included descriptive statistics, independent samples chi-squared test, analysis of variance (Mann-Whitney U test), and multivariate logistic regression analysis, performed using IBM SPSS Statistics v26 (IBM Corp., Armonk, USA).

Results: The study involved 386 participants with a mean BMI of 31.29.29 ± 13.27 kg/m^2^; 46.6% were obese. The overall prevalence of major depressive disorder (MDD) was 34.5%. MDD was significantly more common among younger individuals (18-25 years), female participants, singles, those with university degrees, and individuals earning less than 4000 SR monthly (p<0.05). It was also more prevalent among participants without prior mental health diagnoses and those not using weight-loss supplements. Although MDD was more common among obese individuals, BMI was not a significant risk factor. Multivariate analysis identified low income and use of weight-loss supplements as significant factors associated with MDD (p<0.05).

Conclusion: Our study did not find a significant link between BMI and depression in Al-Madinah Al-Munawwarah, contrasting with some previous research across Saudi Arabia. These findings may be influenced by socioeconomic factors such as gender, marital status, and education level. Future research should consider larger sample sizes within specific age groups to better understand the various contributors to MDD.

## Introduction

Obesity and depression are two serious health issues that have drawn more attention recently. Millions of people worldwide suffer from depression, a mental illness that is a major contributor to disability and is typified by enduring feelings of melancholy and disinterest. The World Health Organization (WHO) states that depression, also known as depressive disorder, is a prevalent mental illness that can affect anyone. This differs from the typical fluctuations in mood and emotions experienced in response to everyday life events [1,2].

Obesity is defined by the WHO as an excessive or aberrant buildup of fat that poses a health concern. Adults are classified as overweight if their body mass index (BMI) is greater than 25 kg/m^2^ and obese if it is equal to or greater than 30 kg/m^2^. Obesity has spread throughout the world, lowering life expectancy and causing a host of chronic illnesses. The co-occurrence of obesity and depression is a complicated problem that needs more research [1-4].

Even though the link between depression and obesity is becoming more well-acknowledged, many questions remain unanswered, particularly in the Al-Madinah Al-Munawwarah region of Saudi Arabia. Although studies have shown that those who are obese have a higher prevalence of depression, more research is required to determine the underlying causes of this association [4-7].

It is crucial to comprehend the connection between obesity and depression for a number of reasons. First of all, people who have these illnesses together may experience worse health outcomes and a lower quality of life. Compared to people who just have one of the conditions, those who have both obesity and depression are more likely to use healthcare services, have less success with therapy, and have a worse prognosis overall [4]. Second, there is a significant financial cost to society, healthcare systems, and individuals. Examining the incidence of depression and the particular difficulties that the obese population faces will help determine the scope of the issue, allocate resources, and develop effective solutions. Last but not least, examining the fundamental processes that connect obesity and depression can aid in the creation of focused preventive and therapeutic approaches, thereby enhancing the general health and standard of living for those who suffer from these illnesses.

This study aimed to assess the association between BMI and depression among the general population in Al-Madinah Al-Munawwarah, Saudi Arabia.

## Materials and methods

Study design, location, and time

A cross-sectional study was conducted in Al-Madinah Al-Munawwarah, Saudi Arabia, from August 2024 to March 2025.

Study population

The inclusion criteria were adult residents ≥18 years of both genders, and the exclusion criteria were residents younger than 18 years and those from other regions of Saudi Arabia.

Sample size

The population of Al-Madinah Al-Munawwarah is approximately 1.6 million [8], and the prevalence of obesity in Saudi Arabia is 24.7% [9,10]. The prevalence of depression among obese individuals is 33.8% [11]. Using a 95% confidence interval (CI) and a margin of error of 5%, the sample size of this study was calculated to be 385 participants according to the Raosoft sample size online calculator (Raosoft Inc., Seattle, USA; www.raosoft.com/samplesize.html).

Sampling technique

A convenience sampling technique was used. This sample technique was selected because it was simple to use, easy to research, and facilitated data collection in a short period of time.

Data collection

Data were collected through an online questionnaire distributed via social media platforms (see Figures 3-9 in the Appendices; Figures 10-12 depict the English translation). Information about participants’ demographic characteristics, chronic diseases, previous diagnoses of mental health disorders, current medications, history of using pills or supplements for weight loss, as well as weight and height, was gathered. Based on the calculated BMI, obesity was considered when BMI was ≥30 kg/m^2^, overweight when BMI was 25 to 29.9 kg/m^2^, normal weight when BMI was 18.5 to 24.9 kg/m^2^, and underweight when BMI was less than 18.5 kg/m^2^.

Depressed mood and depression severity were assessed using the Arabic version of the Patient Health Questionnaire 9 (PHQ-9) [12]. It had nine questions, including the Diagnostic and Statistical Manual of Mental Disorders (DSM-IV) criteria for depression and the main symptoms of depression, with a scoring range from 0 to 27. The last question pertained to the impact of difficulties experienced on daily life by the participants. Depression severity was categorized as follows: (i) none or minimal depression (scores 0-4), (ii) mild depression (scores 5-9), (iii) moderate depression (scores 10-14), (iv) moderately severe depression (scores 15-19), and (v) severe depression (scores ≥20) [13]. As for a previous Saudi study, the sample prevalence of individuals at risk of major depressive disorder (MDD) was a PHQ-9 cutoff >10 [14].

Ethical considerations

Ethical approval for the study was obtained from the Al-Rayan Research Ethics Committee. Confidentiality and privacy were maintained by encoding data and eliminating personally identifiable information.

Data analysis

Data were statistically analyzed using IBM SPSS Statistics v26 (IBM Corp., Armonk, USA). To investigate the association between the variables, a chi-squared test (χ^2^) was applied to qualitative data, which were expressed as numbers and percentages. Quantitative data were expressed as mean and standard deviation (mean ± SD), where the Mann-Whitney U test was applied for non-parametric variables. Multivariate logistic regression analysis was done to assess factors associated with MDD. Odds ratio was calculated at 95% CI. A p-value of less than 0.05 was regarded as statistically significant.

## Results

Demographic characteristics

This study included 386 participants. Of them, 38.6% were 26-44 years old, 57.3% were male, and 48.7% were married. Almost half (50.3%) had a university education, and 38.6% had a monthly income of 4000 SR. About 19.7% had chronic diseases, 10.6% were previously diagnosed with mental health disorders, 26.4% were on regular medications, and 21% used pills or supplements to lose weight (Table 1).

**Table 1:** Distribution of the participants according to their demographic characteristics and medical history (n=386)

Variable	n (%)
Age (years)	
18-25	144 (37.3)
26-44	149 (38.6)
>44	93 (24.1)
Gender	
Female	165 (42.7)
Male	221 (57.3)
Marital status	
Widow	3 (0.8)
Single	181 (46.9)
Married	188 (48.7)
Divorced	14 (3.6)
Educational level	
Illiterate	1 (0.3)
Primary	6 (1.6)
Middle	6 (1.6)
Secondary	89 (23.1)
Diploma	54 (14)
University	194 (50.3)
Postgraduate	36 (9.3)
Monthly income	
<4000 SR	149 (38.6)
4000-8000 SR	55 (14.2)
8001-10000 SR	50 (13)
>10000 SR	132 (34.2)
Chronic diseases	
No	311 (80.6)
Yes	75 (19.4)
Diagnosed with any mental health disorders	
No	245 (89.4)
Yes	41 (10.6)
Do you regularly take any medication?	
No	284 (73.6)
Yes	102 (26.4)
Ever used any pills or supplements to help you lose weight?	
No	305 (79)
Yes	81 (21)

Weight of the participants

**Figure 1:**
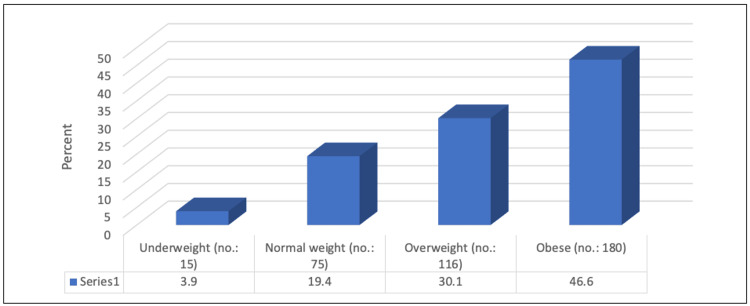
Percentage distribution of studied participants according to BMI categories (n=386) BMI: Body mass index

The mean BMI of the studied participants was 31.29 ± 13.27 kg/m^2^. Based on the BMI classification, 46.6% were obese, 30.1% were overweight, 19.4% had a normal weight, and 3.9% were underweight (Figure 1).

Depression findings

**Figure 2:**
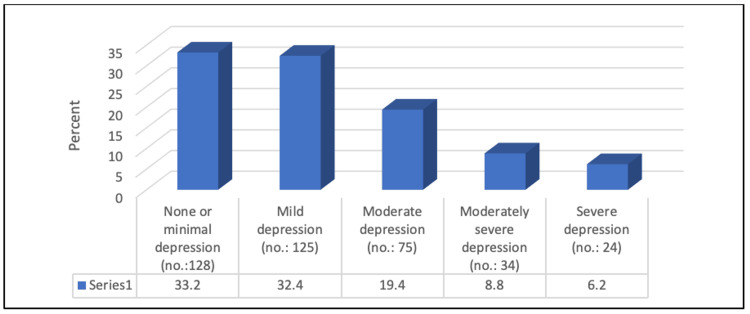
Percentage distribution of levels of depression (n=386)

**Figure 3:**
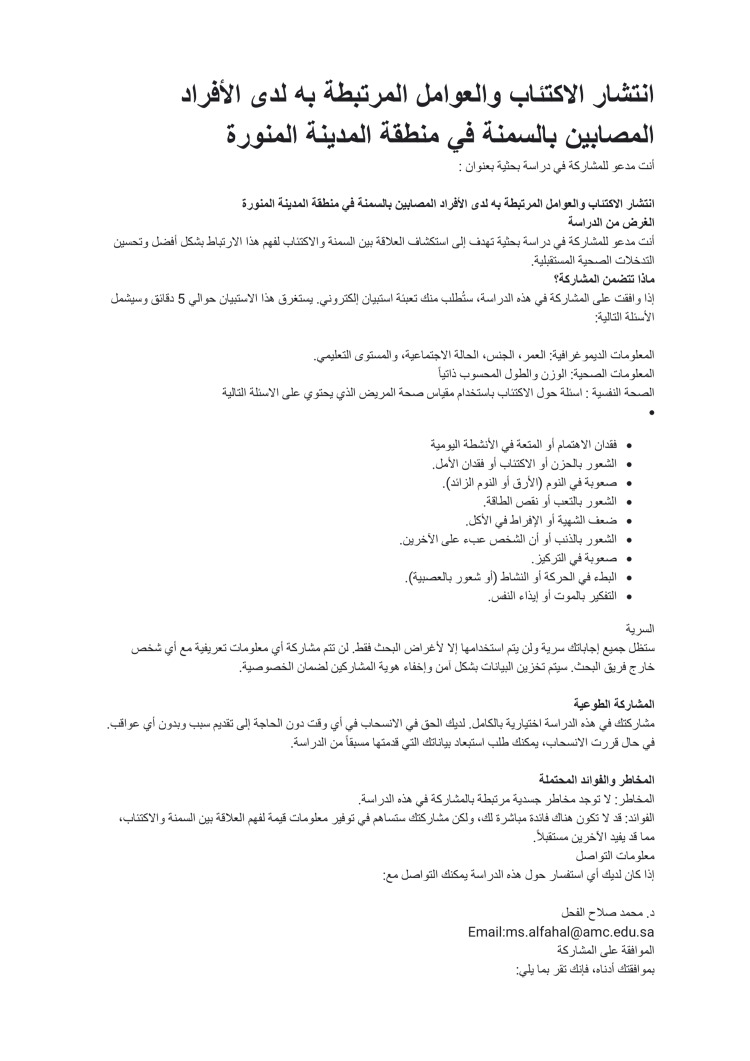
Questionnaire (in Arabic) 1/7

**Figure 4:**
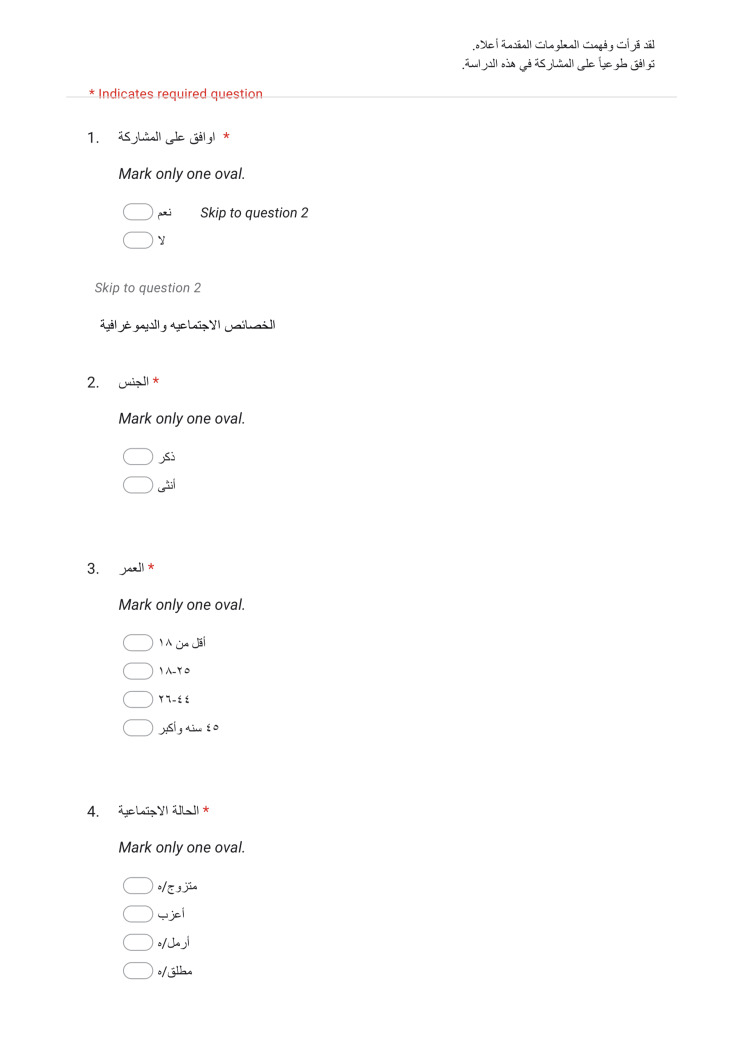
Questionnaire (in Arabic) 2/7

**Figure 5:**
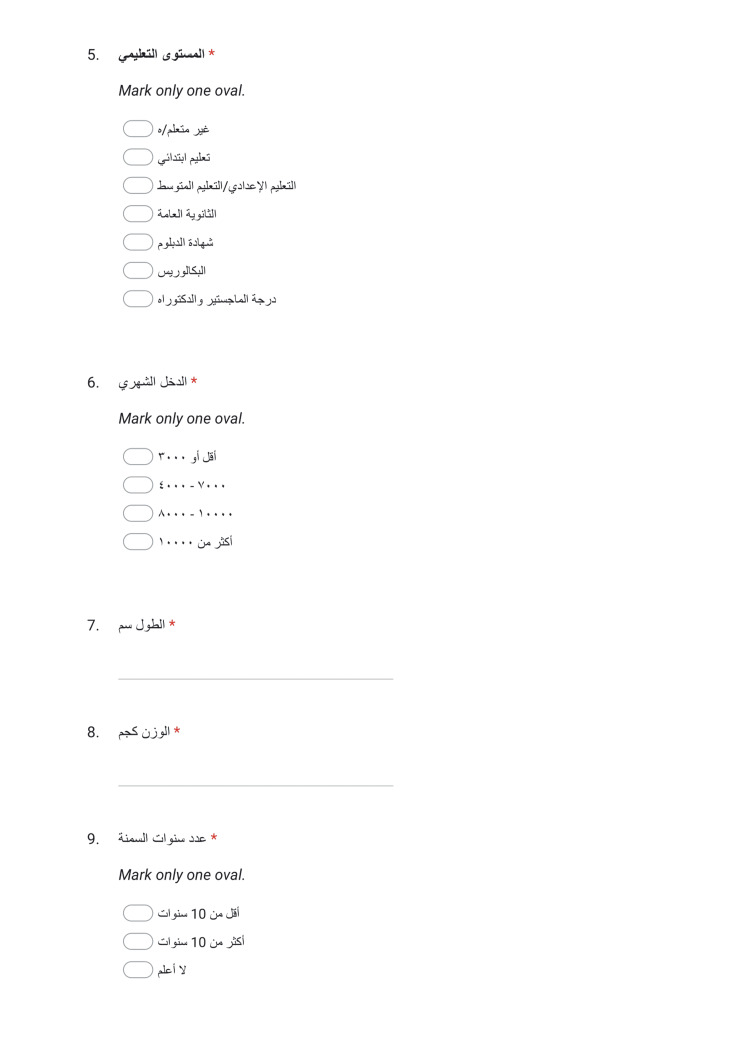
Questionnaire (in Arabic) 3/7

**Figure 6:**
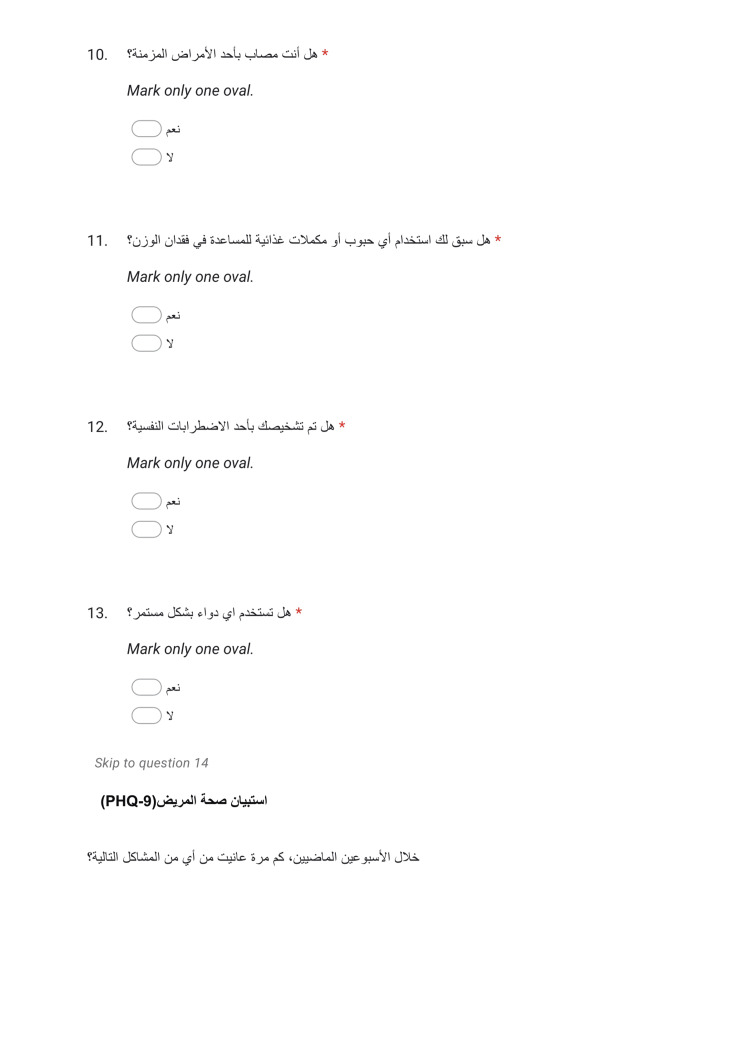
Questionnaire (in Arabic) 4/7

**Figure 7:**
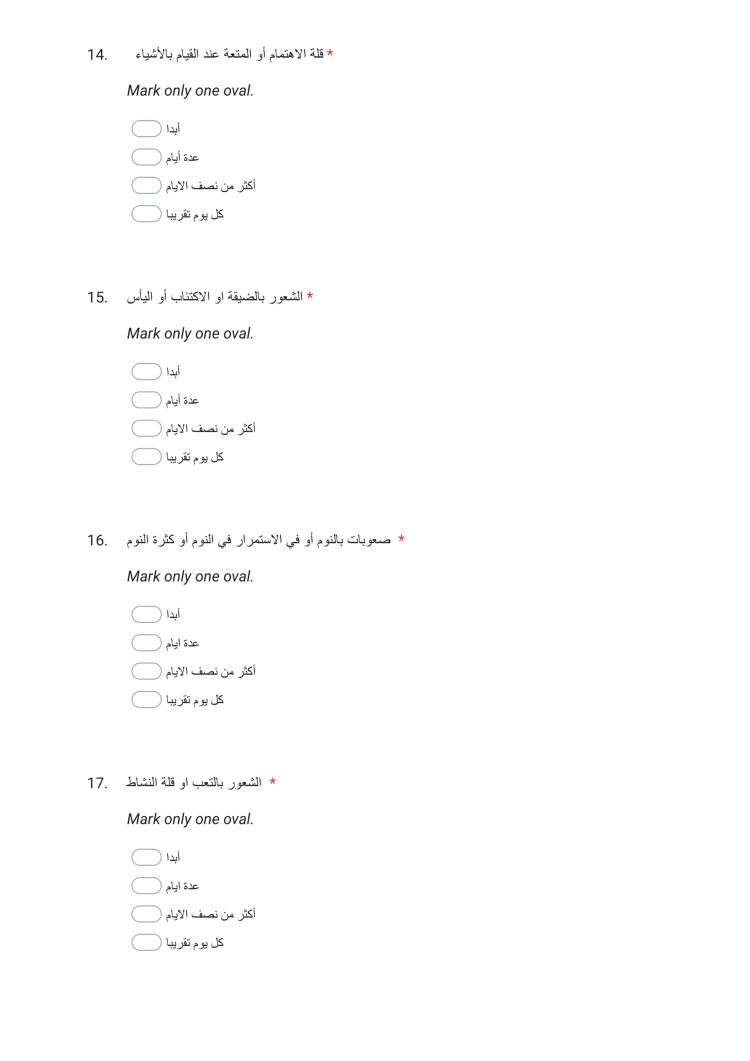
Questionnaire (in Arabic) 5/7

**Figure 8:**
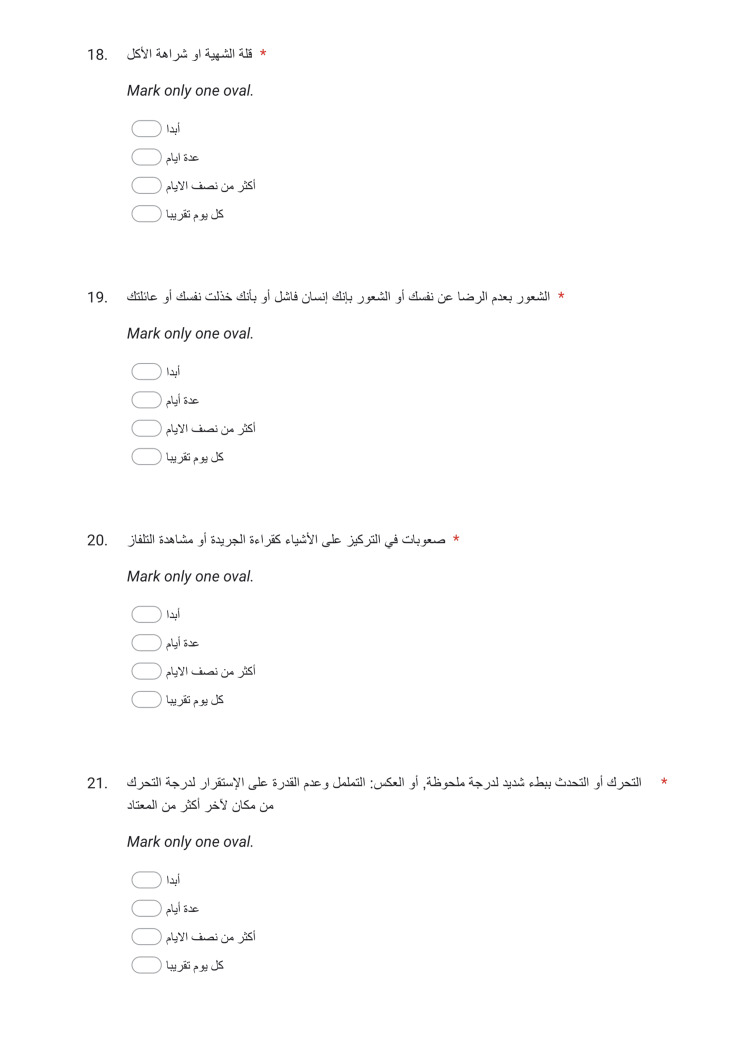
Questionnaire (in Arabic) 6/7

**Figure 9:**
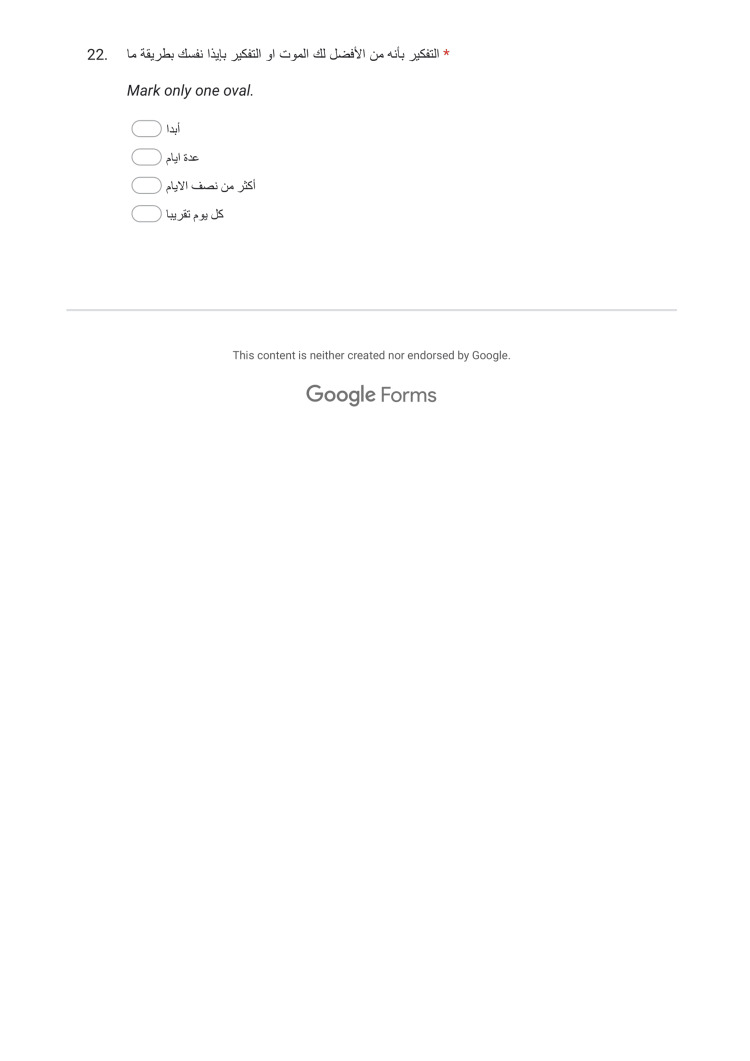
Questionnaire (in Arabic) 7/7

**Figure 10:**
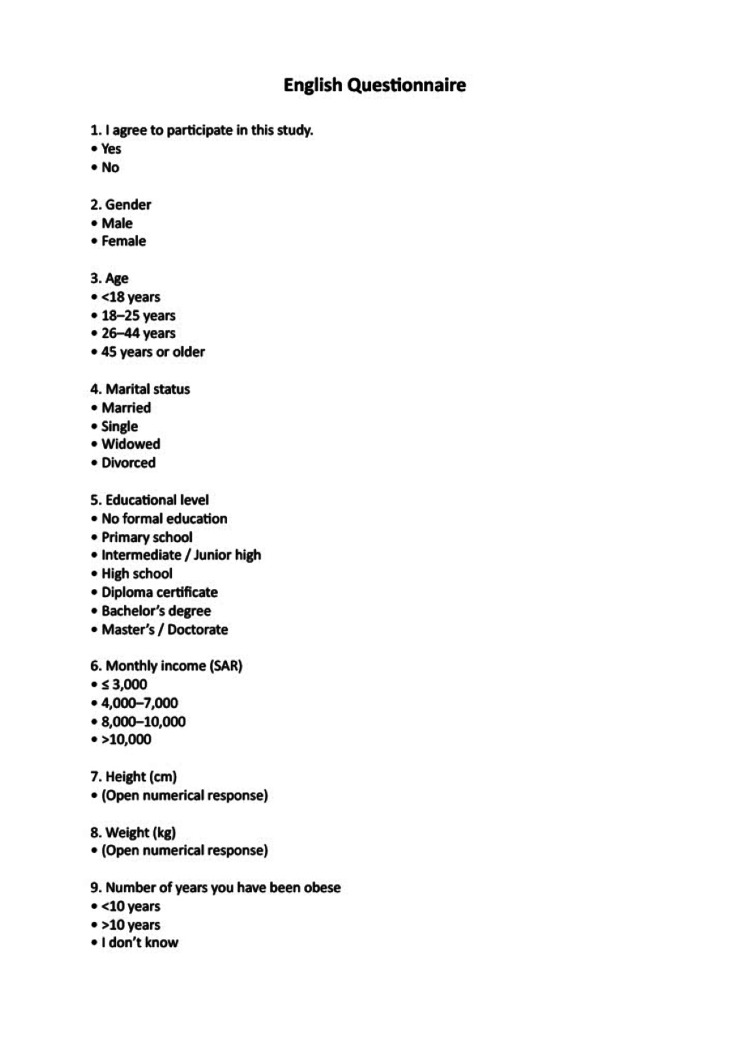
Questionnaire (English translation) 1/3

**Figure 11:**
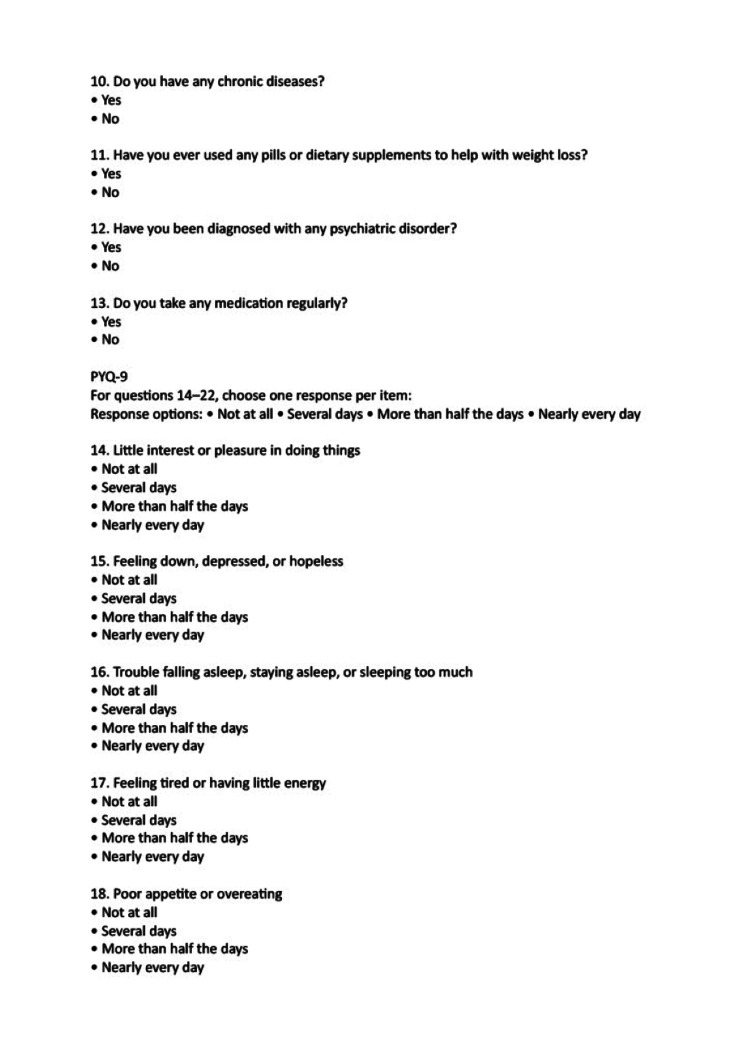
Questionnaire (English translation) 2/3

**Figure 12:**
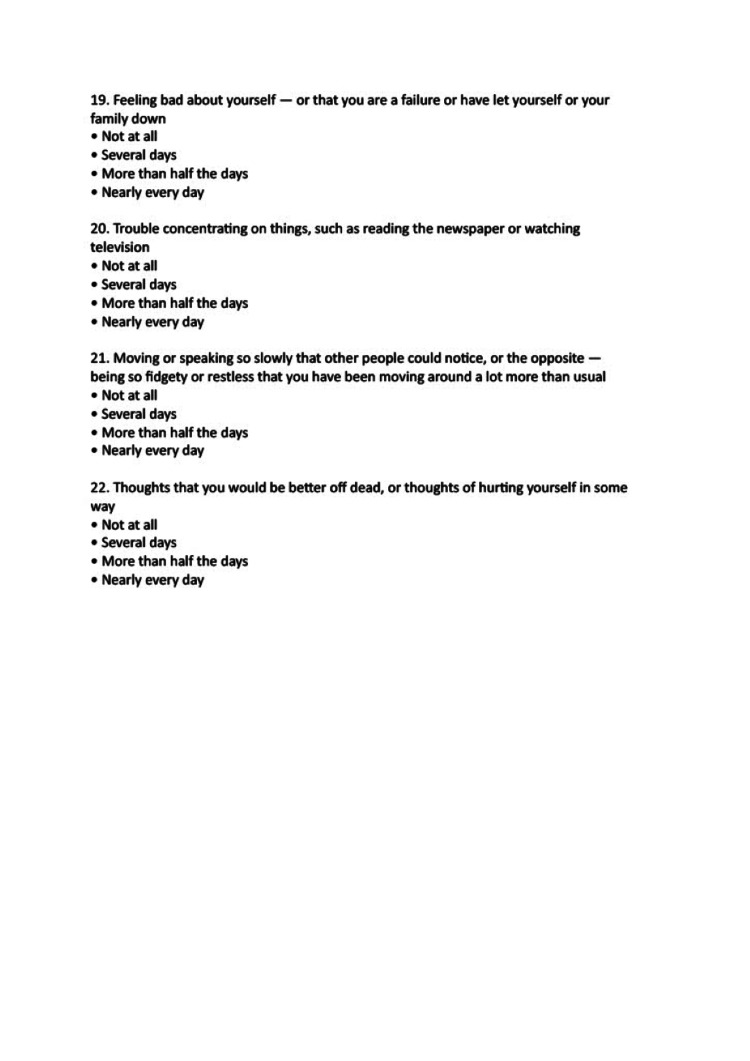
Questionnaire (English translation) 3/3

Figure 2 illustrates that 19.4% of the participants had moderate depression, 8.8% had moderately severe depression, and 6.2% had severe depression. In general, 34.5% were classified as having MDD.

Table 2 shows that the prevalence of MDD was significantly higher among younger participants (18-25 years), female individuals, and those who were single, had a university education, and lowest monthly income (<4000 SR) (p<0.05). At the same time, MDD prevalence was significantly higher among participants with no previous diagnosis of mental health disorders and those who did not use pills or supplements to lose weight (p<0.05). 

**Table 2:** Relationship between the prevalence of MDD and participants’ demographics and medical history (n=386) MDD: Major depressive disorder

Variable	MDD		χ^2^	p-value
	No MDD, n (%)	MDD, n (%)		
Age (years)				
18-25	76 (30)	68 (51.1)	19.34	<0.001
26-44	103 (40.7)	46 (34.6)		
>44	74 (29.2)	19 (14.3)		
Gender				
Female	95 (37.5)	70 (52.6)	8.1	0.004
Male	158 (62.5)	63 (47.4)		
Marital status				
Widow	2 (0.8)	1 (0.8)	13.37	<0.001
Single	96 (37.9)	85 (63.9)		
Married	149 (58.9)	39 (29.3)		
Divorced	6 (2.4)	8 (6)		
Educational level				
Illiterate	0 (0.0)	1 (0.8)	21.17	0.002
Primary	3 (1.2)	3 (2.3)		
Middle	5 (2)	1 (0.8)		
Secondary	42 (16.6)	47 (35.3)		
Diploma	39 (15.4)	15 (11.3)		
University	139 (54.9)	55 (41.4)		
Postgraduate	25 (9.9)	11 (8.3)		
Monthly income				
<4000 SR	76 (30)	73 (54.9)	15.19	<0.001
4000-8000 SR	37 (14.6)	18 (13.5)		
8001-10000 SR	36 (14.2)	14 (10.5)		
>10000 SR	104 (41.1)	28 (21.1)		
Chronic diseases				
No	205 (81)	106 (79.7)	0.09	0.754
Yes	48 (19)	27 (20.3)		
Diagnosed with any mental health disorders				
No	235 (92.9)	110 (82.7)	9.51	0.002
Yes	18 (7.1)	23 (17.3)		
Do you regularly take any medication?				
No	182 (71.9)	102 (76.7)	1.01	0.314
Yes	71 (28.1)	31 (23.3)		
Ever used any pills or supplements to help you lose weight?				
No	212 (83.8)	93 (69.9)	10.11	0.001
Yes	41 (16.2)	40 (30.1)		

Relationship between obesity and MDD

The prevalence of MDD was higher among obese participants compared to those in other BMI categories. Additionally, participants with MDD had a higher mean BMI (30.43 ± 12.31 vs. 32.91 ± 14.85); however, these associations were not statistically significant (p≥0.05) (Table 3).

**Table 3:** Relationship between the prevalence of MDD and BMI categories and mean BMI (n=386) * = χ^2^; ** = Mann-Whitney U test; MDD: Major depressive disorder; BMI: Body mass index

Variable	MDD		Test	p-value
	No MDD, n (%)	MDD, n (%)		
BMI categories				
Underweight	6 (2.4)	9 (6.8)	6.14 *	0.105
Normal weight	52 (20.6)	23 (17.3)		
Overweight	81 (32)	35 (26.3)		
Obese	114 (45.1)	66 (49.6)		
BMI (mean ± SD) (kg/m^2^)	30.43 ± 12.31	32.91 ± 14.85	1.67 **	0.09

High-risk group

Multivariate logistic regression analysis was conducted to identify the risk factors (independent predictors) of MDD among the studied participants. It was found that having a monthly income of less than 4000 SR or ever using any pills or supplements to help lose weight were risk factors (independent predictors) for MDD (p<0.05). In contrast, BMI and prior diagnosis of any mental health disorder were not identified as risk factors for MDD (Table 4).

**Table 4:** Multivariate logistic regression analysis of the risk factors of MDD among the studied patients MDD: Major depressive disorder; BMI: Body mass index

Variable	B	Wald	p-value	Odds Ratio (95% CI)
Age (years)	0.28	1.49	0.222	0.74 (0.47-1.19)
Gender	0.43	3.04	0.081	0.64 (0.39-1.05)
Marital status	0.34	1.57	0.21	0.7 (0.4-1.21)
Educational level	0.12	1.3	0.254	0.88 (0.7-1.09)
Monthly income	0.24	4.67	0.031	0.78 (0.63-0.97)
Chronic diseases	0.36	3.32	0.068	0.69 (0.47-1.02)
Diagnosed with any mental health disorders	0.35	3.69	0.055	0.69 (0.48-1)
Do you regularly take any medication?	0.27	2.28	0.131	1.3 (0.92-1.85)
Ever used any pills or supplements to help you lose weight?	0.89	9.65	0.002	2.44 (1.39-4.29)
BMI	0.02	3.13	0.077	1.02 (0.99-1.04)
BMI categories	0.08	0.27	0.603	0.91 (0.66-1.26)

## Discussion

This study aimed to explore the relationship between BMI and depression among participants selected through convenience sampling in Al-Madinah Al-Munawwarah, located in western Saudi Arabia. We found no significant link between BMI and depression (defined as MDD by a PHQ-9 cutoff >10). Instead, we identified other significant factors contributing to MDD: (i) age (18-25 years), (ii) gender (female), (iii) marital status (single), (iv) educational level (university degree), (v) monthly income (below 4000 SR), (vi) no previously diagnosed mental health disorder, and (vii) having not utilised pills/supplements for weight loss.

Previous studies in Saudi Arabia have focused on the eastern provinces (Almarhoon et al., 2021; Aljabr et al., 2021) [12,15]. In comparison to our findings, the depression burden in our participant population was 33.5%, which is similar to the ~31% reported in primary care settings in Al-Hassa [15]. However, regarding BMI and MDD, our results were in contrast to Almarhoon et al. (2021). In their study of 711 participants, they found a significant association between BMI and moderate to severe depression. Nevertheless, similar to our findings, they reported that being single and aged 18-25 contributed to a higher likelihood of depression [12]. We posit that the difference in our findings pertaining to BMI and depression may be due to their overall larger sample size and a comparatively high proportion of female participants (75%) versus ours (42%), which may be driving an effect. Additionally, societal, economic, and other factors may affect how BMI and depression intersect in the eastern versus the western provinces.

Wider research, such as a national study in Saudi Arabia, found that overall depression was significantly associated with higher BMI [16]. Most participants were from the western region (42.4%), which also had the lowest median depression score (8), compared to the eastern region (9). This, combined with our smaller sample size, could explain why we did not find a significant link between BMI and depression. Finally, unlike our analyses, these authors utilised the Beck’s Depression Inventory-II (BDI-II). This is another validated tool for depression; however, it comprises 21 components and is based on intensity over frequency. Whether this metric is comparable is an important consideration. Another study covering all Saudi regions (Al Shanbari et al., 2024) also gleaned similar findings to Nour et al.’s research: when using PHQ-9, they found a significant correlation between obesity and the highest mean depressive score [16,17]. Both our mean depressive scores ranged close to significance (p=0.09 in Mann-Whitney U test; p=0.077 in multivariate logistic analysis) - perhaps an increase in the number of participants and thus a higher powered study may have resulted in an association.

Studies in China reveal that extremes of BMI (e.g., underweight or obese) contribute to depression (Cui et al., 2024) [18]. This may also be due to the societal pressures of a low body weight, especially in female individuals, contributing to concepts of self-worth, an idea we explore in the following paragraph [19].

A concept named the “jolly fat” hypothesis was coined in 1975, where individuals showed lower rates of anxiety and depression [20]; this led to a later research utilising this concept, which found that in Chinese people aged >45, increased BMI resulted in a decrease in depressive symptoms [21]. Our older age bracket cut-off was 44; this may have resulted in us missing such an effect, or it could be that in this population in western Saudi Arabia, age, BMI, and obesity intersect differently.

With this in mind, we also refer to an Indonesian population, wherein high BMI in adults was negatively associated with depression, with the authors suggesting this may be in part driven by reduced stigma toward being overweight as well as the impact of socioeconomic status [22]. The intersection of globalisation, public perception of obesity, and wealth is an important consideration, as we found that lower income was associated with an increased likelihood of MDD. Indeed, Ali et al. (2024) demonstrated a significant relationship between weight stigma, anxiety, and depression in young adults (average age of 20) in Saudi Arabia [23]. However, no ”jolly fat” effect was present in a German population (Herhaus et al., 2020), again showing that obesity was linked to increased depression (and lower general health) [24].

Finally, in the east of Iran, no meaningful association was found between obesity phenotypes and depression in participants aged over 60, but exercise was found to be protective against depression [25]. As our research included a number of age ranges, this may have diluted the effect of the 18-25 age group contributing to obesity and MDD.

We have discussed age, the potential protective factor of age, the “jolly fat" hypothesis, and societal ideas regarding weight across different countries. Other studies have considered the influence of interventions such as diet and medications [26,27]. These papers suggest that there is a targeted number of individuals, both with obesity and depression, who would yield greater benefits from intervention versus those with only one characteristic. We note in our study that although ~30% of participants had depression, only a small percentage reported taking regular medication. Whether this as an intervention could be valuable to this population warrants further investigation.

Overall, as noted in the literature by Luppino et al. (2010) and Blasco et al. (2020) in their systematic review, the relationship between obesity and depression is complex - depression may contribute to high BMI, and vice versa [28,29]. However, in other individuals, this may result in low BMI (and vice versa). Furthermore, socioeconomic drivers such as the cost of healthy food and lifestyle choices, as well as the perception of body image, may further convolute this picture. We recommend a vital intervention that consists of lifestyle modifications with campaigns to increase public awareness regarding mental health [12,30].

Limitations

The limitations of our study include the use of convenience sampling; this is reflective of the general population in Al-Madinah Al-Munawwarah, Saudi Arabia, and specifically, those who utilise social media (the way in which we disseminated the questionnaire). We used multivariate analysis to take into consideration numerous contributing factors. However, given significant differences, for example, in age and educational level, these factors may compound and reduce the power of our study. Future research should select larger sample populations within certain age brackets to understand the milieu of factors contributing to MDD.

## Conclusions

This study investigated factors linked to MDD in Al-Madinah Al-Munawwarah, Saudi Arabia. Although higher BMI was associated with MDD, the correlation was not statistically significant. MDD was significantly more common among young adults (18-25), female participants, unmarried individuals, university graduates, and those with low income. Remarkably, MDD was also more common among participants who were not previously diagnosed with mental health disorders and those not using weight-loss supplements. Logistic regression analysis identified low income and the use of weight-loss supplements as independent predictors of MDD, whereas BMI was not confirmed as a significant predictor.

These findings underestimate the complex interplay between mental health and socioeconomic determinants. Targeted public health strategies are recommended to address mental well-being, particularly among economically disadvantaged and young adult populations. Future campaigns and interventions should direct attention to improving mental health literacy and addressing the needs of these vulnerable groups.
